# The Development of Response and Interference Inhibition in Children: Evidence from Serious Game Training

**DOI:** 10.3390/children11020138

**Published:** 2024-01-23

**Authors:** Lingyun Wang, Jiajia Li, Fanli Jia, Lin Lian, Lihong Li

**Affiliations:** 1 School of Psychology, Northeast Normal University, Changchun 130024, China; wangly880@nenu.edu.cn (L.W.); lijj073@nenu.edu.cn (J.L.); lianlin@nenu.edu.cn (L.L.); 2 Jilin Provincial Key Laboratory of Cognitive Neuroscience and Brain Development, Northeast Normal University, Changchun 130024, China; 3 Tianjin Vocational Institute, College of Electronical and Information Engineering, Tianjin 300410, China; 4 Department of Psychology, Seton Hall University, South Orange, NJ 07079, USA; fanli.jia@shu.edu; 5 School of Social Welfare, Changchun Humanities and Sciences College, Changchun 130119, China

**Keywords:** inhibitory control, serious game, spatial Stroop task, children

## Abstract

A serious game titled “Crossing the Jungle” was developed in this study to train children’s inhibition skills using the Stroop task. The effects of inhibitory control on children were tested by a pre-test, post-test, and one-month follow-up test. In the control groups, children were asked to play a commercial game instead. In experiment 1, 48 participants chose either the training or control game voluntarily, whereas, in experiment 2, 44 participants were randomly assigned to either group. In both experiments, children exposed to the serious game demonstrated training effects from the Stroop spatial task and near-transfer effects from the Flanker task. However, transferring effects were not produced by the Go/No-go task. As a result, although the serious game “Crossing the Jungle” does not improve response inhibition, children aged 9 to 12 who play it may benefit from improved interference inhibition abilities. This provides evidence for the mutual independence of interference inhibition and response inhibition in children at this stage.

## 1. Introduction

In the modern age of digital technology, electronic devices permeate our daily lives and video games hold a strong allure for children, leading to an increased number of children becoming addicted to gaming [1]. Since video games cannot be completely abandoned, it is important to develop ones that provide entertainment as well as knowledge promotion, skill acquisition, and ability development, which are defined as serious games.

Unlike an electronic game intended for entertainment, a serious game is designed based on principles of computer game design and computer game technology. Serious games are scientific, multimodal, narrative, and exploratory [2] and some have been shown to positively affect cognition, motivation, emotion, and socialization [3]. Researchers have determined that serious games can enhance the cognitive function of people with mild cognitive impairment or Alzheimer’s disease by improving attentional allocation, spatial skills, executive function (EF), and problem-solving skills [4]. Therefore, serious games provide a new method of improving children’s EF, e.g., inhibitory control.

EF refers to an individual’s ability to control and regulate their cognitive processes and behaviors to adapt to constantly changing environments and accomplish various tasks. The components of EF include inhibitory control, working memory, and cognitive flexibility. Inhibition control consists of two main types: response inhibition and interference inhibition/interference control [5,6,7]. One’s level of response inhibition determines whether distractions will prevent them from focusing on a central goal. A study spanning 32 years discovered that children exhibiting higher response inhibition were less prone to indulge in smoking, drinking, or drug use during their teenage years. Additionally, they were more inclined to follow social and moral norms, achieve greater career success, and enjoy happier family lives in their adult years [8]. Similarly, other studies have found that children’s deficient response inhibition can lead to obesity, excessive eating, and substance abuse [9,10,11]. An individual’s level of response inhibition can be measured using Go/No-go and stop-signal tasks. In Go/No-go tasks, individuals are instructed to press a button in response to certain stimuli while refraining from pressing the button when other stimuli are presented; during the stop-signal task, participants must avoid responding when a stop signal appears on a few select trials [5].

Interference inhibition refers to an individual’s ability to suppress distraction from powerful or automatic mental representations of irrelevant information. An individual’s interference inhibition level can be measured using Stroop, Flanker, and Simon tasks. For example, in the classic Stroop color task, an individual must suppress the visual color of color words when reciting them. In the Flanker task, they need to focus on a centrally presented stimulus while disregarding the surrounding stimuli; in the Simon task, individuals are instructed to respond to a particular feature of a stimulus presented on either the right or left side, restraining their natural inclination to react to the stimulus’s location [5]. Individuals with high levels of interference inhibition are able to complete current cognitive activities quickly and efficiently by inhibiting the interference of irrelevant information. As a result, interference inhibition directly affects academic performance, including areas such as reading, spelling, and math [12,13,14].

Children between the ages of three and twelve are in important developmental periods for both forms of inhibitory control. At the age of four, children can suppress simple response inhibition tasks, such as delaying snacking, and can perform complex response inhibition tasks, such as computer Go/No-go tasks, after age eight [15]. By twelve, response inhibition has developed rapidly [16]. Johnstone et al. examined the response inhibition ability of children aged 7–12 by using a stop-signal task and a Go/No-go task. The study found that individuals responded more quickly and accurately as they aged [17]. Furthermore, interference inhibition develops rapidly in children aged three to four. For example, children of this age can complete simple Luria clapping game tasks (i.e., making actions that are inconsistent with the experimenter) but not relatively complex day–night tasks (e.g., saying “night” when seeing a picture of “day”) until after turning five [18]. At approximately eight years of age, children can complete the more difficult Stroop task, and their cognitive abilities continue to develop rapidly during the ages of nine to thirteen [16]. Both inhibitory controls reach adult levels following puberty [19,20]. In summary, the critical period for the development of response inhibition and interference inhibition is childhood. Development in preschoolers (before age 6) focuses on qualitative changes, such as knowing whether or not to inhibit a task. Development in school-age children (after age 6) is more focused on quantitative changes, such as how efficiently an inhibition task is accomplished [15].

Recent research has presented differing views on whether response inhibition and interference inhibition develop independently or simultaneously during childhood. Some researchers used a confirmatory factor analysis (CFA) to solve the issue. Gandolfi et al. (2014) successfully distinguished between response inhibition and interference inhibition in children aged 3–4 and found moderately strong positive correlations, indicating they both developed simultaneously [21]. However, Tiego et al. speculated that the commonality might be attributed to the functioning of working memory capacity, which is a general cognitive capability. They found that when working memory ability was excluded, response inhibition and interference inhibition were structurally independent in 11–12-year-old children [22]. 

Furthermore, whether the effect of response inhibition/interference inhibition training can be transferred to interference inhibition/response inhibition can also provide evidence for the independence of the two types of inhibition control. Unfortunately, most inhibitory control training for children is centered on a comprehensive training program of executive function. Thus, it is difficult to evaluate the transfer effects between response inhibition and interference inhibition separately [23,24]. And a few single-task inhibitory control studies have resulted in contradictory conclusions. The transfer effect between response inhibition and interference inhibition has not been observed in some studies. For example, Liu et al. trained children aged 4–6 to play Go/No-go games without observing an increase in interference inhibition during a Stroop-like task [6]. Zhao, Chen, and Maes used the Go/No-go task to train 10–12-year-old children and adults on response inhibition, but they did not find a transfer effect to the Stroop task of interference inhibition [25]. In contrast to the previous studies, Zhao and Jia used a Stroop-like paradigm to train children aged 10–12. They discovered that children could produce interference inhibition on both the Stroop and the Flanker tasks after training. However, there was a tendency to transfer on the Go/No-go task, reflecting response inhibition (the transfer effect was marginally significant) [26].

The inconsistencies may be due to the suppression training scheme used. Liu et al. utilized a commercial game called “Fruit Ninja” for training purposes [6]. Although the game is similar to the response inhibition task, Go/No-go, its primary purpose was to provide entertainment and leisure. Because many irrelevant factors were mixed into the guidance and training, the training lacked a theoretical basis and thus its validity was reduced. Other researchers developed a more scientific and theoretical computer-based training program [25,26]. Their training tasks were repeated many times during the training process, and the training was mainly conducted at fixed times and locations; however, the degree of freedom of time and space during the training process was restricted. In addition, due to the monotonous training mode, the program did not meet the needs of children who were easily distracted and bored. The evidence for varying training effects due to fluctuations in children’s motivation remains sparse. Therefore, it is important to create a training program that blends enjoyment and scientific elements while addressing the issues posed by commercial game training and computer-based programs. On one side, the newly developed training program should offer efficient techniques to promote the growth of children’s inhibitory skills. On the other side, the program needs to showcase a dependable transfer between the two inhibitory processes.

In the present study, we developed a serious game based on the Stroop task in order to assist children aged 9–12 in improving their interference inhibition. We also investigated whether interference inhibition and response inhibition are independent by examining the training and transfer effects of interference inhibition training from the serious game. Our study involved children aged 9–12 because interference inhibition, which is developing in this age group [15], is closely related to their academic performance [12,13,14].

To overcome the problems in training-based commercial games and computer programs, the serious game was designed in terms of the standardized Stoop task to ensure its theoretical and scientific validity. In addition, the serious game incorporated auxiliary factors such as the player’s role and storyline to enhance children’s training interest. Considering both external and internal validity, as well as the overall training time and the length of each training session impacting training and transfer effectiveness [27,28], this study incorporated two experimental validation phases. The first experiment used a quasi-experimental design where children and parents chose to be part of either the control group or the training group. Three weeks of long-term training were conducted three times a week for fifteen minutes each. The second experiment employed a true experimental design in which children were randomly assigned to a training group or a control group which underwent short-term training for 8 days. We expected that children’s interference inhibition would be more enhanced after serious game training compared to the control group. We also tested whether children’s response inhibition would be enhanced.

## 2. Game Design

### 2.1. Character Setting

The game utilizes third-person perspective. The player controls a brave child hero who must carry out a mission to increase children’s interest and immersion. As the game begins, the character enters a foggy jungle. A castle lies deep within the jungle, and the player’s friends are trapped inside. The player must pass through many levels within a limited time period to reach the castle in the misty forest. The level contains ten obstacles, including blue stones, grass, vines, poisonous mushrooms, wooden barrels, and rose bushes. Obstacles are randomly distributed and divided equally into two groups: consistent obstacles and non-consistent obstacles. Players must respond differently to each obstacle type—consistent obstacles must be dodged according to the normal avoidance method, whereas non-consistent obstacles must be dodged according to the opposite avoidance method. By overcoming all obstacles along the way and passing the level successfully, the player will be able to rescue his friends and receive a mysterious gift bag.

### 2.2. Game Parameter Setting

The game consists of five levels. In different levels, the time interval between the appearance of obstacles and their response decreases as the number and difficulty of levels increases. Previous studies have shown that the average response time for children aged 5–8 in a spatial Stroop task is 700–800 milliseconds; the average response time for children aged 9–11 is 500–600 milliseconds; and the average response time for children aged 12–15 is 400–500 milliseconds [29]. As a general rule, the shorter the reaction time, the more difficult the activity becomes. Throughout the first to fifth levels, the time interval between when an obstacle appears and when a key is pressed is 1500 ms, 1000 ms, 800 ms, 600 ms, and 400 ms. Each level contains 100 obstacles, and the player is required to press the key at each obstacle. If a player fails to press the key, a crash sound will be generated. The player successfully passes the level when reaching an 85% correct rate, allowing them to move on to the next, more difficult level. If a player scores less than 85%, they must restart the level and try again.

### 2.3. Game Reward Mechanism

The game is designed with a competitive element. The game can be played by multiple players simultaneously online. After each level has been completed, the player’s competition ranking is displayed using a star rating system, and the top three players receive varying amounts of gold coins. When a player successfully crosses an obstacle, they receive a gold coin reward, which can be exchanged for character equipment in the game store. The program also provides encouraging words at the end of each level, such as “You are awesome” and “Keep going”.

### 2.4. Gameplay

Before beginning each level, the player is presented with a description of the type of obstacle and a demonstration of how to avoid it. Players should avoid obstacles by following a normal avoidance response in order to achieve consistency. For example, press “→” to move to the right to avoid obstacles on the left, press “←” to move to the left to avoid obstacles on the right, press “↑” to jump over obstacles below, and press “↓” to slide down to avoid obstacles above. In contrast, the reverse obstacle in the game requires players to react oppositely to the normal situation. For example, press “←” to move to the right to avoid obstacles on the left, press “→” to move left to avoid obstacles on the right, press “↓” to jump to avoid obstacles below, and press “↑” to slide to avoid obstacles above. The level is set up with a buffer zone (a scene without obstacles during which the player does not need to perform any operations), which allows the player to take a break. In accordance with the spatial Stroop paradigm, a player must overcome interference from spatial orientation information on button judgment during the game to score points.

## 3. Game Training Test

### 3.1. Experiment 1: Test Based on Quasi-Experimental Design

#### 3.1.1. Participants

We displayed recruitment posters in a primary school that contained an explanation of the research objectives, process, methods, and potential benefits of participating in the research. Upon gaining understanding and permission from parents, students have the option to choose whether to join the experimental group or the control group, and their parents must sign an informed consent form. There was not a designated quota or cap for the number of participants in each selection. We simply accommodated all those who registered for each group. A total of 46 children aged 9–12 participated in the experiment. Out of these, there were 22 children (13 girls and 9 boys; with an average age of 10.22 ± 1.41) who partook in serious game training. The remaining 24 children were assigned to the control group (12 boys and 12 girls; with an average age of 10.29 ± 1.57). It is important to note that all participants stated that they were right-handed, had normal corrected vision, were not colorblind, and had no familial history of mental illness. Following each session of the experiment, participants were given the option to either receive a bonus of 10 Chinese yuan or an equivalent small prize.

#### 3.1.2. Instruments and Equipment

The measurement tasks were programmed and recorded through E-prime 2.0. This program was presented on a 19″ Dell desktop computer with a 1920 × 1080 resolution and a 60 Hz refresh rate. Participants completed the tasks in a quiet, comfortable environment with their eyes about 60 cm from the computer screen.

#### 3.1.3. Research Procedure 

Before initiating the training program, we assessed the inhibitory control levels of both groups of children. After the pre-test, children in the training group played the custom-designed game “Cross the Jungle”, while those in the control group played a common game “Talking Tom Parkour”. The training occurred thrice weekly for three weeks, with each session lasting approximately 10–15 min. All children completed the game sessions during their free time at home, with their parents supervising and collaborating. A research assistant supported parents by pre-installing games on electronic devices and sending regular reminders about the training sessions. A post-test was administered immediately following the training period, and a subsequent follow-up test was conducted one month later.

#### 3.1.4. Measurement Tasks

The spatial Stroop task was used to measure the level of interference inhibition and evaluate the training effect of serious games. Each trial started with a fixation point “+” in the middle of the screen. After 250 ms, the “+” was replaced by an arrow pointing to the left or right. The subject’s task was to press the corresponding key and then a blank screen would appear for 1000 ms. Based on the correspondence between the direction of the arrow and the position on the screen, the trials were divided into three types: consistent trials (arrows pointing and presenting in the same position), neutral trials (arrows appearing in the center of the screen), and inconsistent trials (arrows pointing and presenting differently). The task consisted of 20 practice trials and 100 experimental trials. The ratio of consistent trials, inconsistent trials, and neutral trials was 2:2:1, and the order of trials was randomly distributed. Average response times for different trial types with response times >200 ms and correct responses were recorded. The mean response time of non-congruent trials was subtracted from the mean response time of congruent trials as an indicator. The larger the value, the weaker the interference inhibition ability.

The Flanker task was used to evaluate the effect of near transfer—that is, the effect of interference inhibition. Each trial was first presented with the fixation point “+” for 500 ms, followed by a blank screen for 500 ms, a stimulus screen for 1500 ms, and a blank screen for 1000 ms, and then followed by a second trial. The stimulus screen contained five arrows, where the middle arrow was the target and the remaining four arrows were the distractors. The player’s task was to judge the direction of the target arrow. The trials consisted of congruent trials (the central and bilateral stimuli pointed in the same direction) and inconsistent trials (the central and bilateral stimuli did not point in the same direction). The task consisted of 16 exercises and 128 formal tests (a 1:1 ratio of concordant to discordant trials, presented randomly). Average response times for different trial types with response times >200 ms and correct responses were recorded. The mean response time of discordant trials was subtracted from the mean response time of concordant trials as an interference indicator. Higher values indicated weaker interference inhibition.

A Go/No-go task was used to assess response inhibition (i.e., the effect of distant migration). At the beginning of each trial, a fixation point “+” was presented in the center of the screen for 1000 ms, followed by the letters “X or Y” for 1600 ms, and then followed by a blank screen for 1000 ms. In the first two blocks, participants were required to press the “J” key when an “X” appeared on the screen and to not respond when a “Y” appeared. The latter two blocks required participants to press the “J” key when a “Y” appeared on the screen and to not respond when an “X” appeared. The ratio of Go/No-go trials in each block was 1:1, presented randomly, and subjects were required to complete the task as quickly and accurately as possible. The task consisted of 20 practice trials and 200 formal trials. The mean reaction time of Go trials and the discriminative power d’ of all trials were used as indicators to assess response inhibition. The larger the d’ value, the stronger the reaction inhibition ability.

#### 3.1.5. Results

**Figure 1:**
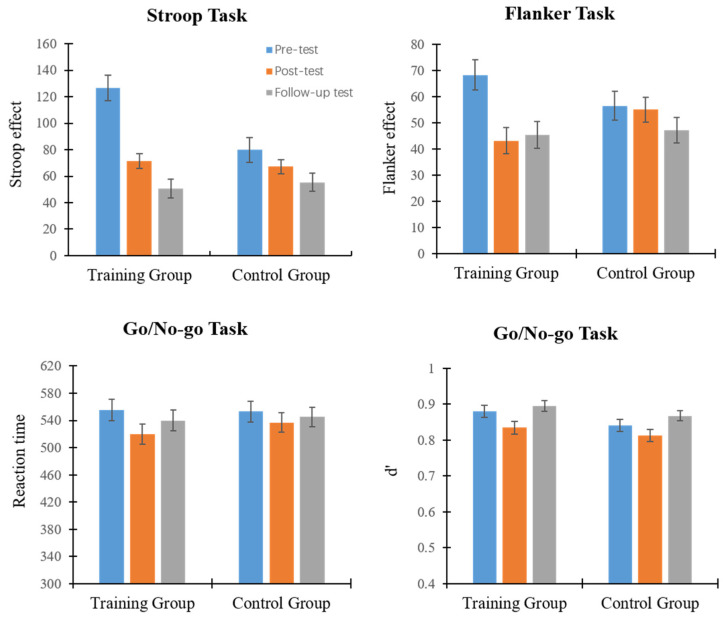
Mean score of the measurement of each task during pre-test, post-test, and follow-up test in the training group and the control group of exp.1.

The pre-test, post-test, and follow-up test data for the training and control group are presented in Figure 1. A repeated ANOVA of 2 (group: training group or control group) × 3 (measurement time: pre-test, post-test, or follow-up test) was assessed for the dependent variables in the Stroop task, Flanker task, and Go/No-go task, respectively.

There was a significant main effect of group on the spatial Stroop task: F (1, 44) = 5.05, *p* = 0.03, and ηp^2^ = 0.103. The interference score in the training group was significantly higher than the interference score in the control group, which indicates that the children in the training group had a significantly lower level of interference inhibition than those in the control group. The main effect of measurement time was significant: F (2, 88) = 26.78, *p* < 0.001, and ηp^2^ = 0.378. A post hoc analysis revealed that the pre-test scores were significantly higher than those of the post-test and follow-up test (*p*s < 0.001), and post-test scores were significantly higher than follow-up scores (*p* < 0.01). The interaction between measurement time and group was significant: F (2, 88) = 7.71, *p* = 0.001, and ηp^2^ = 0.149. Simple effect analysis found that the children in the training group had a significantly higher pre-test interference score than in the post-test and follow-up-test (*p*s < 0.001), and the post-test interference score was significantly higher than the follow-up test score (*p* = 0.019), indicating that serious game training effectively reduced interference inhibition and could be maintained for one month. In the control group, there was no significant difference in the scores between the pre-test, post-test, and follow-up test (*p*s > 0.107). The pre-test scores were marginally higher than the post-test scores (*p* = 0.056), indicating that other game training and time effects had some effect on the spatial Stroop task of children in the control group. We also compared the Stroop effect between the training group and the control group, respectively, in the pre-test, post-test, and follow-up test and found that the Stroop effect of the training group was significantly higher than that of the control group in the pre-test (*p* < 0.01), but there were no significant differences between the two groups in the post-test (*p* = 0.583) and follow-up test (*p* = 0.638), indicating that the interference control level of the training group and the control group were not equal at baseline.

In the Flanker task, the main effect of group was not significant, but the main effect of measurement time was significant: F (2, 88) = 6.98, *p* = 0.002, and ηp^2^ = 0.137. The results of a post hoc analysis showed that interference scores from the pre-test were significantly higher than scores from the post-test and follow-up test (*p* = 0.01); however, interference scores from the post-test and follow-up test were not significantly different (*p* = 0.526). The interaction between group and measurement time was significant: F (2, 88) = 3.32, *p* = 0.041, and ηp^2^ = 0.070. Simple effect analysis revealed that for children in the training group, the pre-test interference score was significantly higher than those of the post-test and follow-up test (*p* < 0.01), and no significant difference existed between the post-test and follow-up test (*p* = 0.736). There were no significant changes (*p*s > 0.154) in the control group for all three measurements. We also compared the inference scores between the training group and the control group, respectively, in the pre-test, post-test, and follow-up test and found that there were no significant differences between the two groups in the pre-test (*p* = 0.150), post-test (*p* = 0.090), and follow-up test (*p* = 0.793).

On the Go/No-go task, the results showed no significant effect of group, nor an interaction between group and measurement time. The main effect of measurement time was significant: F (2, 88) = 4.62, *p* = 0.012, and ηp^2^ = 0.10. Following post hoc comparisons, it was found that the reaction time for the Go trial was significantly lower in the post-test (*p* = 0.010). However, no significant differences were found between the pre-test, post-test, and follow-up tests (*p*s > 0.095). Both groups of children demonstrated an improvement in reaction speed over time when performing the Go/No-go task. The repeated measures analysis of variance for d’ showed that the main effect of group was not significant: F (1, 44) = 2.34 and *p* = 0.133. In the control group, the main effect of measurement time was significant: F (2, 88) = 15.71, *p* < 0.001, and ηp^2^ = 0.263. In the post hoc analysis, there was a marginally significant difference between the follow-up test and the pre-test (*p* = 0.051), whereas the measurement time in the follow-up test was significantly higher than in the post-test (*p*s < 0.001). There was no significant interaction between measurement time and group. The Go trial reaction time and discriminative d’ results consistently showed that children in both the training and control groups had improved performance on the Go/No-go task, possibly due to time effects or multiple repeated measures.

### 3.2. Experiment 2: Based on True Experimental Design

Except for the experimental subjects and the research process, the designs of the game were the same as experiment 1.

#### 3.2.1. Participants 

We randomly recruited 48 children aged 9–12. Four did not complete the experiment, and the other forty-four valid subjects were randomly assigned to either the training group or the control group. There were 22 children in the training group (10 girls and 12 boys; mean age 10.05 ± 1.13), and 22 children in the control group (11 girls and 11 boys; mean age 10 ± 1.03). All participants were right-handed, had corrected to normal vision, were not colorblind, and had no family history of mental illness. Parental consent was obtained before the experiment began through a signed informed consent form.

#### 3.2.2. Research Process

All participants were pre-tested and randomly divided into groups before training. Children in the serious training group and the control group conducted game training in the school computer room in a uniform manner. The children in the training group played the serious game developed for this study called “Through the Jungle” for 10 min a day for 8 days. At the same time, the children in the control group played a commercial game called “Talking Tom Parkour”. A post-test was conducted right after the training and a follow-up test was conducted one month later.

#### 3.2.3. Analysis of Results

**Figure 2:**
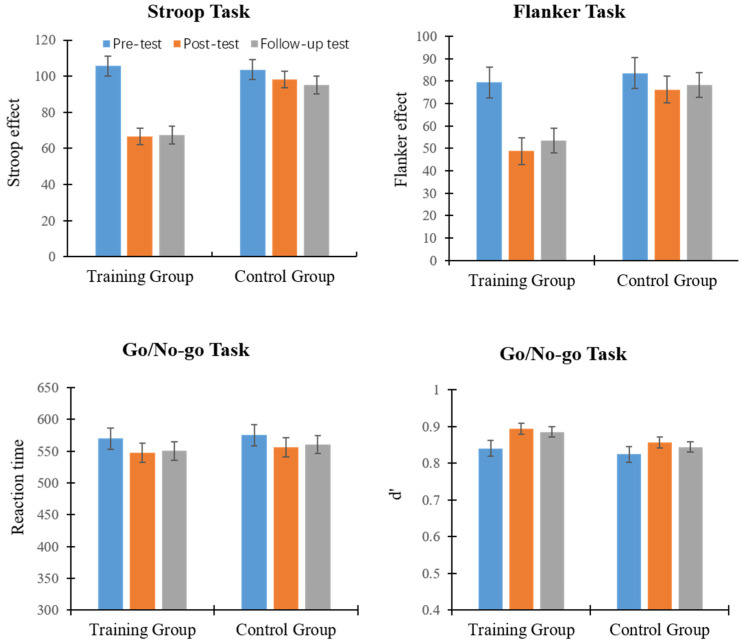
Mean score of the measurement of each task during pre-test, post-test, and follow-up test in the training group and the control group of exp.2.

The training and control group data from the pre-test, post-test, and follow-up test are shown in Figure 2. Similar to experiment 1, the dependent variables in the Stroop task, the Flanker task, and the Go/No-go task were, respectively, 2 (group: training group or control group) × 3 (measurement time: pre-test, post-test, or follow-up test) repeated measures analysis of variance. 

The results of the Stroop task data showed that the main effect of measurement time was significant: F (2, 84) = 48.31, *p* < 0.001, and ηp^2^ = 0.54. Post hoc tests found that the interference scores for the post-test and follow-up test were significantly lower than those for the pre-test (*p*s < 0.001). There was no significant difference between the post-test and follow-up test scores (*p* = 0.611). The main effect of group was significant: F (1, 42) = 9.04, *p* = 0.004, and ηp^2^ = 0.18. Children in the training group had significantly lower interference scores than those in the control group. The interaction between measurement time and group was significant: F (2, 84) = 23.49, *p* < 0.001, and ηp^2^ = 0.36. Simple effect analysis showed that the pre-test interference scores of children in the training group were significantly higher than those of the post-test and follow-up test (*p*s < 0.001). There was no significant difference between the post-test and follow-up test interference scores (*p* = 0.828), indicating that serious game training effectively reduced the interference score, and that the training effect continued for one month. In the control group, there was no significant difference between the pre-test and post-test, nor between the post-test and follow-up test (*p*s > 0.128). The pre-test scores were marginally higher than those of the post-test (*p* = 0.057), showing some variation in interference inhibition after repeated measurements. Moreover, there was no significant difference between the training group and the control group in the pre-test (*p* = 0.801), and the interference scores of the children in the control group were significantly higher than those in the training group in the post-test and follow-up test (*p*s < 0.001). As the above data indicate, children who participated in serious game training had significantly lower interference scores on the spatial Stroop task compared with the control group, which indicates that there is a significant training effect, and that the training effect continues for one month following the training.

In the Flanker task, there was a significant main effect of measurement time: F (2, 84) = 34.01, *p* < 0.001, and ηp^2^ = 0.45. The post hoc test showed that the pre-test interference score was significantly higher than in the post-test and follow-up test (*p*s < 0.001), and there was no significant difference between the post-test and follow-up test scores (*p* = 0.134). The main effect of group was significant: F (1, 42) = 5.29, *p* = 0.027, and ηp^2^ = 0.11, and the interference scores in the control group were significantly higher than those in the serious game training group. The interaction between group and measurement time was significant: F (2, 84) = 13.63, *p* < 0.001, and ηp^2^ = 0.25. Simple effect analysis found that the children in the training group had a significantly higher interference score in the pre-test than in the post-test and follow-up test (*p*s < 0.001), yet there was no significant difference between the post-test and follow-up test scores (*p* = 0.144). Pre-test and post-test scores of the children in the control group differed marginally (*p* = 0.070), and there was no significant difference between their pre-test and post-test scores or their post-test and follow-up test scores (*p*s > 0.126). There was no significant difference in the scores of children in the training and control group in the pre-test (*p* = 0.672), and the interference scores of the children in the training group were significantly lower than those in the control group in the post-test and follow-up test (*p*s < 0.01). In sum, the children in the training group significantly improved their performance in the Flanker task after serious game training. Since the effect was maintained for a month, the serious game training migrated to the Flanker task as well.

In the Go/No-go task, repeated measures analysis of variance showed that neither the main effect of group nor the interaction between group and measurement time was significant. The main effect of measurement time was significant: F (2, 84) = 6.01, *p* < 0.01, and ηp^2^ = 0.13. The post hoc test found that the action time of the Go trial was significantly higher in the pre-test than in the post-test (*p* < 0.001) and in the follow-up test (*p* = 0.024). There was no significant difference between post-test and follow-up test action time (*p* = 0.667), suggesting that the level of response inhibition increased in both groups, possibly as a result of multiple measurements rather than serious game training. The repeated measures analysis of variance for d’ found that the main effect of group was not significant—F (1, 42) = 1.95 and *p* = 0.170—yet the main effect of measurement time was significant: F (2, 84) = 17.24, *p* <0.001, and ηp^2^ = 0.29. The post hoc test found that the measurement times during the post-test and follow-up test were significantly higher than during the pre-test (*p*s < 0.01), and there was no significant difference between the post-test and the follow-up test (*p* = 0.092). The interaction between measurement time and group was not significant: F (2, 84) = 1.55, *p* = 0.218. The results indicated that there was an improvement in the levels of response inhibition in both groups of children; however, the training effect of serious games did not transfer significantly to this task. 

## 4. Discussion

The purpose of this study was to design and develop the serious game “Through the Jungle” based on the space Stroop task and to verify the training and transfer effects of the serious game through two experiments. Both experiments found that children in the training group had a training effect on the Stroop task, a near-transfer effect on the Flanker task, but no transfer effect on the Go/No-go task.

In the two experiments, the reaction time scores of the children in the training group were significantly reduced in the Stroop task, which indicates that serious game training improved children’s interference inhibition ability in the spatial Stroop task and produced a training effect. This result is consistent with Zhao and Jia’s computer program training results [26]. The Stroop task can be effectively improved after a certain period of training with the same interference inhibition principle.

The serious game was designed in accordance with the spatial Stroop task. Similar to inconsistent trials in the spatial Stroop task, the game participant must avoid inconsistent obstacles and prevent spatial information from influencing judgment. By playing serious games, children become more adept at inhibiting interference information and making correct responses, improving their ability to inhibit interference. In contrast to Zhao and Jia’s purely theoretical study, the current study incorporated the fun element in addition to theory, purpose, and relevance, allowing children to show enthusiasm and interest in the training, which in turn maintains children’s motivation for training at a relatively stable level and produces more convincing results [26]. Consequently, we may be able to design more types of serious games in the future to improve the executive function of individuals.

Both experiments showed that the children in the training group had distraction scores on the Flanker task that were significantly lower after serious game training, indicating that the training effect of serious games on the spatial Stroop task resulted in a significant near transfer to the Flanker task, consistent with others’ results [25,26]. As both the Stroop and Flanker tasks are used to assess interference inhibition ability, there may be a similarity in the cognitive processing between these tasks, as the participants are required to filter distractions and respond correctly. Furthermore, neuroscience research has shown that both the Stroop and Flanker tasks activate brain regions such as the right dorsolateral prefrontal cortex (right DLPFC) and the anterior cingulate cortex (ACC) during the response selection phase [30]. Compared with the Flanker task, the distraction in the Stroop task is more difficult to suppress because it requires more attention resources from individuals. As a result, the inhibitory training effect on the Stroop task is more likely to be observed on the relatively simple Flanker task. Thus, similar tasks are easier to transfer. As demonstrated by Wang et al.’s study, adolescents can transfer their stop-signal training to the Go/No-go task, which is also a response inhibition task [27].

Neither experiment found that the training effect of the Stroop task transferred to the Go/No-go task for the children in the training group. This contrasts with the results of Zhao and Jia, who found that interference inhibition has a marginally significant transfer effect on response inhibition [26]. Similar to the current study’s findings, Wilkinson and Yang found that elderly individuals who repeated training on the Stroop task were able to improve their performance on the Stroop task, but not on the Go/No-go task [31]. Furthermore, researchers who trained preschool children or healthy adults using the Go/No-go task did not observe a transfer effect of response inhibition training on children’s Stroop tasks [6,32]. These results demonstrate that training effects based on interference inhibition or response inhibition may not be transferable, which provides indirect evidence that the development of the two inhibitory controls occurs at relatively independent stages in school-age children.

We conducted a correlation analysis on the pre-test scores for the three tasks in each experiment to further explore the possibility of transference. We found only high correlations between the scores of the Stroop task and the Flanker task (r of experiment 1 = 0.320, *p* < 0.05; r of experiment 2 = 0.389, *p* < 0.01), which may indicate that the Stroop task and the Flanker task reflect the same ability, whereas the Go/No-go task reflects a different ability. The Stroop task utilizes the access function of inhibition, which requires participants to avoid focusing their attention on irrelevant interference information. As a type of response inhibition, the attempt to control automatic behavioral responses falls under the behavioral category [5,33]. According to studies of relevant brain mechanisms, response inhibition activates the same brain regions as interference inhibition in addition to the basal ganglia, which is associated with motor initiation and termination and the supplementary motor area/pre-motor area (SMA/pre-SMA), which is involved in forming and updating motor plans [34]. Accordingly, the above findings provide empirical support that response inhibition and interference inhibition are two distinct inhibitory mechanisms. 

Two experiments were conducted in this study to examine the effect of serious game training. Children who voluntarily chose the training group were found to have low interference inhibition control in experiment 1. The level of interference inhibition significantly improved after serious game training, and the training effect persisted for one month after the training. This result supports the compensation model, which suggests that the effect of cognitive training is affected by the individual’s baseline level, and individuals with low baseline levels have greater plasticity and room for improvement [24,35]. In experiment 2, children were randomly assigned to the training or control group. Children in both groups demonstrated middle to high levels of interference inhibition. After the serious game training, the children’s interference inhibition levels were significantly improved. The results of this study support previous research on cognitive training on individual inhibition where a significant improvement in control was also observed [26,31,36].

In previous studies, the duration of inhibitory control training ranged from 5 days to 5 weeks, and the duration of each training ranged from 5 min to 45 min [6,24,36,37]. This study employed two different training schedules in experiment 1 and experiment 2, resulting in significant training and near-transfer effects. The training time was shortened in experiment 2, and short-term serious game training was also effective in improving children’s interference inhibition levels, consistent with the findings of Liu et al. [6] 

## 5. Conclusions

Based on the results of the two experiments, it can be concluded that serious game training can improve the interference inhibitory ability of children with low and normal levels of interference inhibition, and the short and long-term training were equally effective. But the training effects of interference inhibition did not transfer to response inhibition. The results suggest that interference inhibition and response inhibition develop independently in children aged 9–12. 

## 6. Limitations

The serious game developed in this study showed cognitive advantages for children, representing a significant effort to implement serious games for executive function training. Nevertheless, this study faced certain limitations. Firstly, despite our efforts to enhance the game’s scene, screen layout, 3D landscapes, and character representation, it still lacks refinement compared to popular commercial games available today. Secondly, while we utilized quasi-experimental and true experimental designs to assess the serious game’s effectiveness, the small sample size and heterogeneous age groups warrant future research with larger randomized controlled trials to further investigate how individual differences in pre-existing control abilities and varying ages influence participants’ gains from serious game training. Thirdly, our study did not evaluate the distal transfer effects of interference inhibition training through such games on other executive functions like working memory and cognitive flexibility or its subsequent impact on fluid intelligence. Lastly, although the children involved in our research were typically developing individuals, we assume that serious game training might also benefit those with clinical impairments of response inhibition or executive functioning, including children with ADHD. Future research should explore these topics more thoroughly.

## Data Availability

The data presented in this study are available on request from the corresponding author. The data are not publicly available due to privacy restrictions and intellectual property protection.
